# A negative loop within the nuclear pore complex controls global chromatin organization

**DOI:** 10.1101/gad.264341.115

**Published:** 2015-09-01

**Authors:** Manuel Breuer, Hiroyuki Ohkura

**Affiliations:** The Wellcome Trust Centre for Cell Biology, School of Biological Sciences, University of Edinburgh, Edinburgh EH9 3JR, United Kingdom

**Keywords:** chromatin, nuclear pore, meiosis, *Drosophila*, karyosome

## Abstract

The nuclear pore complex (NPC) tethers chromatin to create an environment for gene regulation, but little is known about how this activity is regulated to avoid excessive tethering of the genome. Here we propose a negative regulatory loop within the NPC controlling the chromatin attachment state, in which Nup155 and Nup93 recruit Nup62 to suppress chromatin tethering by Nup155. Depletion of Nup62 severely disrupts chromatin distribution in the nuclei of female germlines and somatic cells, which can be reversed by codepleting Nup155. Thus, this universal regulatory system within the NPC is crucial to control large-scale chromatin organization in the nucleus.

## Abstract (precis)

Breuer and Ohkura propose a negative regulatory loop within the nuclear pore complex (NPC) controlling the chromatin attachment state, in which Nup155 and Nup93 recruit Nup62 to suppress chromatin tethering by Nup155.

The nuclear pore complex (NPC) is the molecular hub for transport in and out of the nucleus. The NPC contains ∼30 nucleoporins that are organized into distinct subcomplexes, including a core scaffold, peripheral nucleoporins, and central channel porins with characteristic phenylalanine–glycine (FG)-rich repeats (Alber et al. 2007). Embedded in the nuclear envelope, the NPC serves as a selective barrier to control nucleo–cytoplasmic, bidirectional transport (Floch et al. 2014).

Ever since a physical association of the NPC with the genome was postulated (Blobel 1985), increasing evidence has pointed to a role for the NPC in tethering chromatin to create an environment for gene regulation. Tethering specific genomic loci to the NPC appears to contribute to transcriptional activation (Casolari et al. 2004; Taddei et al. 2006; Light et al. 2010; Ikegami and Lieb 2013; see Pascual-Garcia and Capelson 2014). Also, the NPC has been further implicated in creating a repressive environment (Green et al. 2012; Van de Vosse et al. 2013) or retaining genes at the periphery after repression, possibly contributing to epigenetic transcriptional memory (Light et al. 2010). However, specific gene regulation modules aside, little is known about how the association of chromatin at a global scale is regulated to avoid excessive, unwanted attachment and how misregulation affects chromatin organization.

Here we propose that the NPC contains a regulatory circuit controlling the chromatin attachment state in the female germline and somatic cells. Loss of an NPC subunit, Nup62 or Nup93, leads to excessive chromatin attachment to the nuclear envelope, which can be rescued by codepletion of a chromatin-binding NPC subunit, Nup155. This study highlights a major role of the NPC in global chromatin organization and suggests a universal regulatory system within the NPC.

## Results and Discussion

### NPC subunits Nup62 and Nup93 suppress excessive chromatin attachment to the nuclear envelope

**Figure 1.**
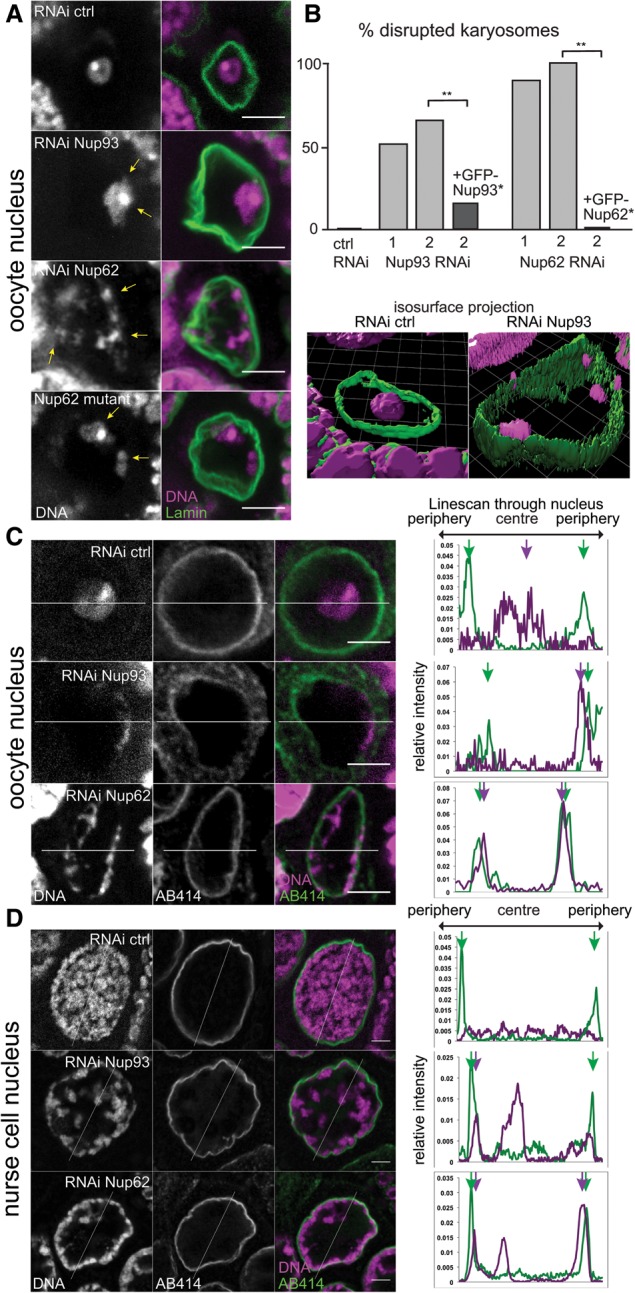
Excessive chromatin positioning at the nuclear periphery upon loss of Nup93 or Nup62 in oocytes and nurse cells. (*A*) The karyosome in the oocyte nucleus is proximal to the nuclear periphery (arrows) upon *Nup93* or *Nup62* RNAi or a *Nup62Δ95* mutant. Bar, 5 µm. (*B*) Frequencies of disrupted karyosomes in oocytes expressing a control shRNA or shRNA against *Nup93* (column 1 or 2; nonoverlapping shRNA) or *Nup62* (column 1 or 2) and in oocytes expressing shRNA2 and a GFP-tagged transgene resistant to RNAi (*). (**) *P* < 0.0001. *n* = 35 for control; 27 ≤ *n* ≤ 50 for *Nup93*; 18 ≤ *n* ≤ 47 for *Nup62*. (*C*,*D*) Maximum intensity projections of three midsections (total 1.5 µm) of oocyte (*C*) or nurse cell (*D*) nuclei expressing control shRNA or shRNA against *Nup93* or *Nup62* with relative signal intensities along a line. Arrows indicate peaks of DNA and nuclear pore signals (AB414).

Cytological study of the chromatin attachment state to the nuclear envelope is experimentally challenging, as chromatin usually occupies the entire nucleus. However, meiotic chromatin becomes fully detached from the nuclear envelope and compacted into a spherical structure, the karyosome, after recombination in *Drosophila* oocytes (Fig. 1A; King 1970). Chromatin detachment and karyosome formation are crucial to make a single spindle and allow subsequent chromosome segregation (Cullen et al. 2005) and are conserved features also seen in mammalian oocytes (Parfenov et al. 1988). By taking advantage of this unique nuclear organization in oocytes, we sought factors required for chromatin detachment from the nuclear envelope by individually knocking down various nuclear proteins in the female germline (the oocyte and nurse cells) by RNAi.

Strikingly, the depletion of either of two nuclear pore proteins, Nup62 or Nup93 (Supplemental Fig. S1A), led to disruption of the compact karyosome morphology (Fig. 1A,B), while the depletion of several other pore proteins did not (Supplemental Fig. S2). The chromatin shifted near the nuclear periphery, resulting in strong (Nup62) or partial (Nup93) overlap with a nuclear pore marker in the oocytes in comparison with the control RNAi (Fig. 1C), which was confirmed by superresolution microscopy (Supplemental Fig. S3). Nup93 is a linker scaffold protein known to be required for the recruitment of Nup62, one of the central channel proteins containing FG repeats (Grandi et al. 1993, 1995; Sachdev et al. 2012). We confirmed that the defect is not an off-target effect by rescue experiments using RNAi-resistant transgenes (Fig. 1B). In addition, similar karyosome defects were observed in female sterile *Nup62* mutants that we generated (Fig. 1A; Supplemental Fig. S4A–C). In nurse cells (polytenized germline cells that support oocyte growth), chromatin also distributed irregularly and more toward the nuclear periphery after RNAi of these genes (Fig. 1D). This demonstrates a general role for both genes in global chromatin organization rather than being restricted to oocytes.

### Excessive chromatin attachment is independent of the meiotic checkpoint and is only associated with mild transport defects

To identify the cause of the karyosome defect upon Nup62 or Nup93 RNAi, we first tested the structural integrity and transport function of NPC. RNAi of Nup62 or Nup93 did not disrupt the overall structural integrity of the NPC, as judged by the localization of FG-containing subunits and the core scaffold subunit Nup107 (Supplemental Fig. S5A–C). The active import function of the NPC showed small differences as assessed by fluorescence recovery after photobleaching (FRAP) of GFP fused with a nuclear localization signal (NLS) (Supplemental Fig. S5D). There was a significant increase in the nuclear size of early oocytes (Supplemental Fig. S5E), which may be caused by a reduced ability of the nuclear pore to act as a diffusion barrier.

Next, we examined a relationship with the meiotic recombination checkpoint, which is known to disrupt karyosome formation in the presence of unrepaired double-strand breaks (DSBs) in oocytes (Ghabrial and Schüpbach 1999). Inactivation of the checkpoint did not suppress the karyosome defects of Nup62 or Nup93 RNAi (Supplemental Fig. S4D), demonstrating that the defect is independent of the meiotic recombination checkpoint in oocytes.

### NPC-interacting chromatin is enriched at the nuclear periphery upon Nup93 depletion

**Figure 2.**
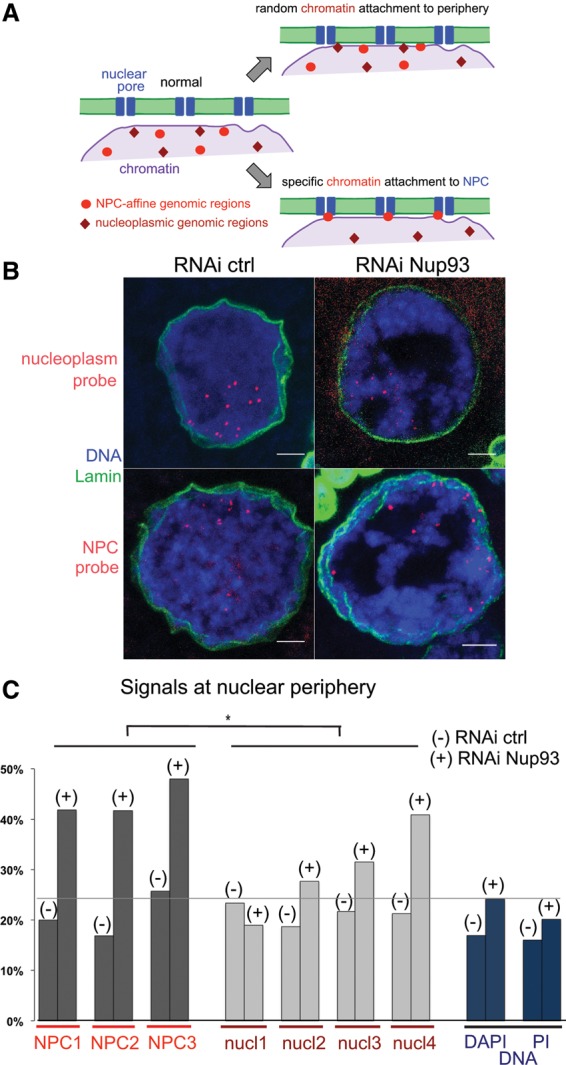
NPC-interacting chromatin is more frequently located at the nuclear periphery without Nup93. (*A*) Nuclear pore-interacting genomic regions found in S2 cells migrate more closely to the nuclear periphery than nucleoplasmic genomic regions upon Nup93 depletion if the pores excessively tether chromatin. (*B*) FISH signals for known NPC-bound regions were more frequently located at the nuclear periphery than those for nucleoplasmic regions when Nup93 is depleted as compared with a control. Bars, 5 µm. (*C*) Percentages of FISH and DAPI/propidium iodide (PI) signals in the nuclear periphery (the distance from the nuclear lamina is <10% of the nuclear radius) in nurse cells subjected to control and *Nup93* RNAi. (−) Control RNAi; (+) *Nup93* RNAi. 79 ≤ *n* ≤ 203. Changes from control RNAi to *Nup93* RNAi were significantly greater for the three NPC probes than the four nucleoplasmic probes. (*) *P* = 0.0179.

Considering the above results, we hypothesized that chromatin is excessively anchored to the NPC in RNAi of Nup62 or Nup93. If this was the case, we predicted that chromatin specifically interacting with the NPC must be preferentially accumulated at the nuclear periphery rather than random chromatin (Fig. 2A). In order to test this, we used previously identified genomic loci bound to another nuclear pore component, Nup98, in *Drosophila* S2 culture cells (Kalverda et al. 2010). Nup98 has two distinct populations—one at nuclear pores and the other in the nucleoplasm—that bind distinct genomic loci in S2 cells (Kalverda et al. 2010). Nurse cells were subjected to fluorescence in situ hybridization (FISH) using individual probes corresponding to genomic loci known to be associated with Nup98 within the NPC or located in the nucleoplasm in S2 cells and were costained with a DNA dye (Fig. 2B). We measured a proportion of the total DNA signals in the nuclear periphery region, defined by a distance from the nuclear lamina of <10% of the nuclear radius, which occupies ∼20% of the nuclear area. In control RNAi, ∼16%–17% of the total DNA (propidium iodide or DAPI signal) was located in the nuclear periphery region (Fig. 2C). For all genomic loci (three NPC-bound and four nucleoplasmic), 17%–25% of the signal foci were found in the nuclear periphery (Fig. 2C). This indicates that there is no preference for periphery locations of the total DNA or of these specific genomic regions in wild-type nurse cells. When Nup93 was knocked down, there was a small increase (from 16%–17% to 20%–24%) in the total DNA that occupies the nuclear periphery (Nup93 RNAi was used, since it gives a milder phenotype than Nup62 RNAi) (Fig. 2C). Strikingly, we observed a strong, consistent redistribution of all NPC-bound genomic loci to the periphery (from 17%–25% to >40%), whereas the nucleoplasmic loci showed smaller variable changes (Fig. 2C). The increases for NPC-bound loci were significantly higher than the increases for both total DNA and the nucleoplasmic loci, supporting our hypothesis that depletion of Nup62 or Nup93 results in an excessive attachment of specific chromatin regions to the NPC.

### Nup155 is required for chromatin attachment to the nuclear envelope in the absence of Nup62 or Nup93

**Figure 3.**
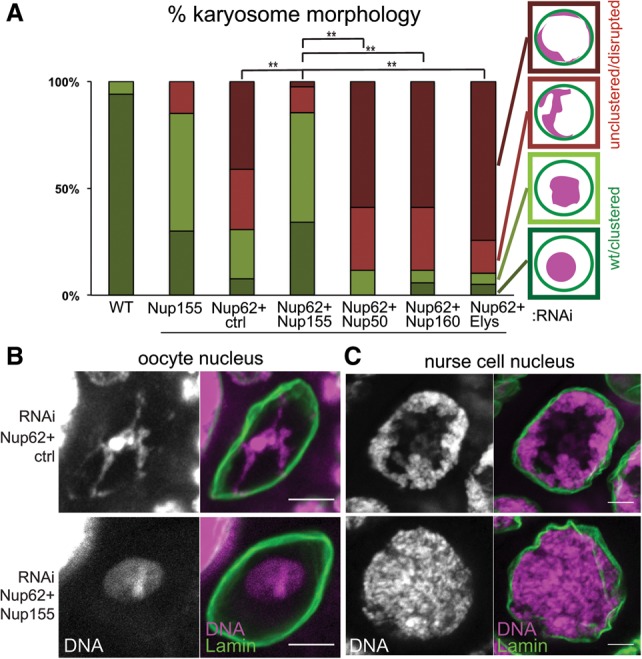
Codepletion of Nup155 rescued excessive chromatin attachment to the nuclear periphery caused by Nup62 depletion. (*A*) Karyosome morphology was classified into four categories, from normal to severely defective (diagrams at the *right*). For double RNAi of control + *Nup62*, *Nup155* + *Nup62*, and *ELYS* + *Nup62*, 39 ≤ *n* ≤ 41; for double RNAi of *Nup50* + *Nup62* and *Nup160* + *Nup62*, *n* = 17. The combined proportion of the two severe categories in *Nup62* + *Nup155* double RNAi was significantly lower than in others. (**) *P* = 0.0001. (*B*,*C*) Restored chromatin morphology in the oocyte (*B*) and nurse cell (*C*) nuclei upon *Nup62* and *Nup155* double RNAi in comparison with *Nup62* and control RNAi. Bar, 5 µm.

Our results suggest that Nup62 and Nup93 suppress the interaction between chromatin and another NPC subunit. If this is the case, codepletion of this hypothetical NPC subunit that mediates chromatin attachment to the nuclear pore should restore detachment of chromatin in Nup62- or Nup93-depleted oocytes. Several NPC subunits have previously been shown to have chromatin-binding activity, including Nup155, Nup50, and ELYS/Mel-28 (Gillespie et al. 2007; Kalverda et al. 2010; Busayavalasa et al. 2012). We generated flies expressing two shRNAs: one for Nup62 and the other for each of the aforementioned chromatin-binding NPC subunits, the non-chromatin-bound Nup160, or a control (Fig. 3A; Supplemental Fig. S1B). Codepletion of Nup155 specifically restored normal karyosome morphology and detachment from the nuclear periphery in Nup62-depleted oocytes (Fig. 3A,B). Furthermore, in nurse cells, simultaneous RNAi of Nup155 also restored normal chromatin distribution caused by Nup62 RNAi (Fig. 3C). Crucially, codepletion of Nup155 did not rescue the larger nuclear size in Nup62-depleted oocytes. This demonstrates that Nup62's function on chromatin organization is independent of its function on nuclear size maintenance (Supplemental Fig. S6A), which may reflect its function as a diffusion barrier.

We also found that single depletion of Nup155 led to a large reduction of Nup62 (one of the FG-containing subunits) from the nuclear envelope and its accumulation in the cytoplasm. However, it did not significantly reduce the total amount of the FG-containing subunits at the nuclear envelope in both Nup155 and Nup62/Nup155 double RNAi (Supplemental Fig. S6B–D). This demonstrates that Nup155 is required for Nup62 recruitment, and the apparent rescue of the Nup62 depletion defect by Nup155 codepletion is not due to a loss of integrity or a reduced number of nuclear pores. Taken together, the results suggest a negative regulatory loop in which Nup155 recruits Nup62 to the nuclear pores, and, in turn, Nup62 suppresses chromatin anchoring by Nup155.

### Somatic cells harbor a common regulatory circuit that controls chromatin distribution

**Figure 4.**
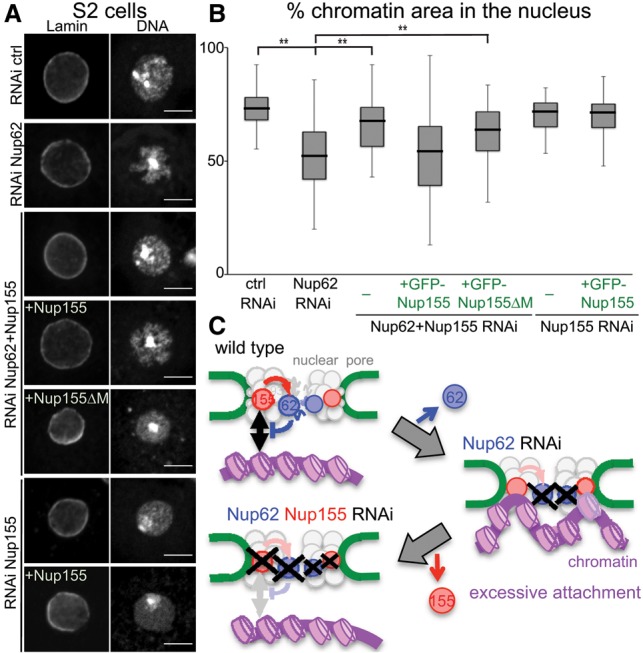
Loss of Nup62 disturbs chromatin distribution in somatic cells, which is rescued by codepletion of Nup155. (*A*) RNAi-treated (control, *Nup62*, *Nup155*, or *Nup62* + *Nup155*) S2 cells were immunostained for Lamin and DNA. Some batches transiently expressed GFP-tagged RNAi-resistant full-length Nup155 (Nup155) and Nup155 lacking the chromatin-binding region (Nup155ΔM). Bar, 5 µm. (*B*) The proportion of the area occupied by chromatin compared with the entire nuclear area (Lamin). The box represents the middle two quartiles, and the whiskers represent the top and bottom quartiles. 23 ≤ *n* ≤ 48. (**) *P* < 0.01, a significant difference from Nup62 RNAi. (*C*) Model for the chromatin attachment state controlled by an internal regulatory circuit in the NPC.

We uncovered a potential negative regulatory circuit within the NPC that controls the chromatin attachment state to the nuclear pores in the oocytes and nurse cells. Therefore, we sought to test whether a common regulatory system also controls chromatin organization in somatic cells. Using the *Drosophila* S2 cell line, we depleted Nup62 **(**Supplemental Fig. S6E) or Nup155 individually and simultaneously by RNAi. Control RNAi cells showed a relatively even distribution of chromatin within the nucleus except for a dense region that corresponds to heterochromatin (Kellum et al. 1995). In contrast, Nup62 RNAi resulted in an uneven distribution of chromatin within the nucleus (Fig. 4A). To quantify this, we measured the area that chromatin occupies relative to the nuclear area. The cells depleted of Nup62 showed a significant decrease in chromatin occupancy compared with a control RNAi (Fig. 4A,B). Strikingly, double depletion of Nup62 and Nup155 showed a chromatin occupancy similar to the control (Fig. 4A,B). This rescue was reversed by RNAi-resistant full-length Nup155 but not by resistant Nup155 lacking the chromatin-binding region (Fig. 4A,B; Busayavalasa et al. 2012). No significant change in chromatin occupancy was observed upon Nup155 depletion alone (Fig. 4A,B). This demonstrated the presence of a common negative loop within the NPC that controls the global chromatin distribution between female germline cells and somatic cells.

### A negative loop within the NPC controls global chromatin organization

Recent reports described the role of the NPC to tether chromatin and thus create an environment for gene regulation (Taddei et al. 2006; Brown et al. 2008; Light et al. 2010; Kalverda et al. 2010; Ikegami and Lieb 2013; Van de Vosse et al. 2013; Pascual-Garcia and Capelson 2014). While recruitment mechanisms for specific genes have been described (Rodríguez-Navarro et al. 2004; Schmid et al. 2006; Light et al. 2010; Van de Vosse et al. 2013), very little is known about whether or how this tethering is regulated. This study makes two major conceptual advances in our understanding of global chromatin organization, especially the critical role and regulation of the NPC-mediated tethering. First, it highlights a far greater role of the NPC in large-scale chromatin organization than previously anticipated. Second, it points to a universal regulatory circuit inside the NPC that controls the attachment state of chromatin to the nuclear pore. This consists of a negative regulatory loop in which chromatin-binding Nup155 recruits the central channel protein Nup62, which in turn suppresses chromatin binding (Fig. 4C). As nuclear pore components associate with the genome to positively or negatively influence gene expression (Pascual-Garcia and Capelson 2014; Ptak et al. 2014), this regulatory loop might be part of a wider network for the NPC to control gene expression, depending on the cellular and developmental context. Although a genuine and direct regulatory role of this loop has yet to be demonstrated, its intrinsic capacity supplies the NPC with a key mechanism to globally or locally organize the metazoan genome. On the other hand, any change or imbalance in this regulatory network might have dramatic effects for the nuclear architecture and, concomitantly, the expression profile of the cell. This may have a significant medical implication, as nuclear pore components not only are known to deteriorate with age but are also affected in several tissue-specific human diseases (Cronshaw and Matunis 2004; Capelson and Hetzer 2009; D'Angelo et al. 2009).

## Materials and methods

### *Drosophila* genetics

Standard fly techniques were followed (Ashburner et al. 2005). Flies were cultured at 25°C in standard cornmeal medium. For RNAi in ovaries, *P{Gal4::VP16-nos.UTR}MVD1* or *P{MatTubulin67C-Gal4}V37* flies were crossed with the following RNAi TRiP (Transgenic RNAi Project) lines (Harvard Medical School): *Nup93* (HMS00850 and HMS00898), *Nup62* (GLV21060 and GL01533), *Nup155* (*DmNup154*; HMS01189), *Nup50* (HMS01054), *Nup160* (HMS00385), and *ctrl-attP2* (*P{CaryP}attP2*); *ctrl-w*^+^ (GL00094). *GFP-Nup107* (*P{GFP-Nup107.K}9.1*) (Katsani et al. 2008) and *GFP-NLS* (*P{Ubi-GFP.nls}ID-2; P{Ubi-GFP.nls}ID-3I*) were also used.

*Nup62* mutants were generated by remobilization of P element (*GSV1-GS2186*). The transposase gene Δ*2–3* was crossed in, and chromosomes that had lost the *w*^+^ gene were tested over a deficiency [*Df(2R)BSC550* or *Df(2R)Exel6063*] for viability and female fertility. Viable chromosomes were tested over the deficiency for the presence of the initiation codon of *Nup62* by PCR. Plasmids containing UASp-GFP-Nup93* and UASp-GFP-Nup62* (the asterisk indicates shRNA-resistant) or Walium22-vectors (Harvard Medical School) containing shRNA against *Mel-28/ELYS* were injected into *w* embryos carrying an *attP40* or *VK33* landing site by Genetic Research, Inc., for transgenesis.

The meiotic recombination checkpoint was suppressed by a heterozygous mutation, *mnk*^p6^ (*DmChk2*) (Klattenhoff et al. 2007), or by feeding adults with medium containing spectral ice blue dye (Sugarflair Colours Ltd.) and 1 mg/mL caffeine (Sigma). *spn*^*A1*^ (Tearle and Nüsslein-Volhard 1987) was used as control.

### *Drosophila* S2 cells

Culture of S2 cells and RNAi were carried out as previously described (Dzhindzhev et al. 2005). For RNAi, S2 cells were incubated for 5–7 d with dsRNA against *Nup62*, the control β-lactamase gene, or the 5′ untranslated region (UTR) of *Nup155*. Some cells were transfected 2 d before fixation with a plasmid containing the metallothionein promoter followed by the *Nup155* or *Nup155ΔM* (540–958 amino acids were replaced by SASA) coding sequence.

### Molecular techniques

Standard techniques were used throughout (Sambrook et al. 1989). *Nup93*- and *Nup62*-coding regions were introduced into the Gateway (Invitrogen) entry vector pDONR221 and then into the destination vector φPGW (pPGW carrying the φ*C31 attB* site; UASp-GFP) or pMAL-Gateway (MBP fusion). To create the expression constructs resistant to the shRNAs, silent mutations were introduced (*Nup93*, GACAACTTG > GATAATTTA; *Nup62*, ATCGTCGAG > ATAGTTGAA) using QuickChange XLII site-directed mutagenesis kit (Agilent). To generate a Walium22-based plasmid that expresses shRNA targeting ELYS/Mel-28, the TRiP protocol (http://www.flyrnai.org/supplement/2ndGenProtocol.pdf) was followed using the oligonucleotides CTAGCAGTGCACTGTGCTGTTGGTTGATCTAGTTATATTCAAGCATAGATCAACCAACAGCACAGTGCGCG and AATTCGCGCACTGTGCTGTTGGTTGATCTATGCTTGAATATAACTAGATCAACCAACAGCACAGTGCACTG, respectively.

RT-qPCR was carried out as previously described (Nikalayevich and Ohkura 2015) except three ovary pairs from adult females (matured for 3–5 d at 25°C) were used. *Actin5C* was used as a control for normalization.

### Cytological techniques

Ovaries and S2 cells were immunostained and analyzed according to Lancaster et al. (2007) and Dzhindzhev et al. (2005). Primary antibodies were used as follows: mAB414 (mouse, 1:200–500 for immunofluorescence; Covance/Biolegend) (Aris and Blobel 1989), HP1 and Lamin (mouse C1A9 1:250 for Western blot, and mouse ADL67.10 1:200–250 for immunofluorescence; Developmental Studies Hybridoma Bank), and anti-Nup62 (rat 1:200–500 for immunofluorescence and 1:250 for Western blot) (this study). Nup62 antibodies were raised against MBP-Nup62 purified from *Escherichia coli*, and the final bleed was used. Secondary antibodies (1:15,000) were purchased from Jackson Immunologicals, Molecular Probes, or Odyssey (Li-Cor) and used as detected by an Odyssey scanner (Li-Cor) for Western blot. For live imaging, oocytes were dissected from matured adult females in halocarbon oil (700) and observed using a spinning disc confocal microscope (UltraView, Perkin Elmer). Typically, a series of *Z*-sections (separated by 0.5 µm) was taken for ovarioles. For live DNA visualization, ovaries were washed in medium containing 5 μM DRAQ5 (Biostatus Ltd.) and mounted in oil. Single *Z*-planes or maximum intensity *Z*-projections of selected planes are shown in the figures after contrast was adjusted uniformly across the field using ImageJ (National Institutes of Health). For structured illumination microscopy (SIM), a Nikon N-SIM (Nikon) with a water immersion objective (60×, 1.2 NA; Nikon) and a xIon897 EMCCD camera (the gain at 300; Andor) was used. Images were reconstructed using the Nikon N-SIM software, and the measured resolution in the final image was 160–170 nm. For the reconstruction, the Wiener filter and apodization filter parameters were kept constant for all samples (Gustafsson et al. 2008).

### FRAP experiments

Ovaries expressing shRNA and GFP-NLS were observed in halocarbon oil. A region of interest (ROI) corresponding to the nucleus was drawn on the mid *Z*-plane of the oocyte nucleus (stages 4–6). Images were acquired using laser power 5%, gain 650, and pinhole with 6.2 airy units every 8 sec for 40 time points. After five time points, the ROI was photobleached by 488-nm laser (100% power) with 25 iterations. The average intensity for each oocyte nucleus was corrected using the nurse cell nucleus as a nonphotobleached control. Averages for all nuclei in sample groups were entered into the equation for a fitted curve of expected recovery, and *t*1/2, as time needed to reach recovery equal to one-half maximum recovery, was determined as described (Colombié et al. 2013).

### FISH

The following CH322-based BACs containing the genomic regions that bind NPC-tethered Nup98 and nucleoplasmic Nup98 in S2 cells (NPC1–3 and nucleoplasm1–4) (Kalverda et al. 2010) were used for FISH: 53P20, 169H03, and 176O04 + 12I13 were used for NPC1–3, and 120G02 + 175O14, 70N20, 51D05, and 92H12 were used for nucleoplasmic1–4 (BACPAC Resources Center). BACs were digested with AluI, HaeIII, MseI, MspI, RsaI, and Sau3AI in 4BC buffer + BSA + DTT. DNA was precipitated and resuspended in TE. Ten micrograms of DNA was denatured for 2 min at 95°C and put on ice before labeling with ChromaTide Alexa-564 dUTP and unlabeled dTTP (1:8 ratio) using 60 U of terminal deoxynuleotidyl transferase (TdT) in TdT buffer for 1 h at 37°C. Glycogen was added, and probes were subjected to a Sephadex G-25 column before being precipitated, washed, and resuspended in TE. Oocytes were fixed in prewarmed 3.7% formaldehyde for 4 min and washed in 2× SSCT. Oocytes were gradually put into 2× SSCT/50% formamide and incubated for 2–4 h at 37°C before incubating in 1.1× hybridization buffer with 50–600 ng of the FISH probe (final 40 µL) at 37°C. Samples were subsequently washed out of 2× SSCT/formamide and, after final washes, in 2× SSCT, followed by immunostaining protocol.

### Image analysis

To generate line-scan intensity profiles, images were background-subtracted, and several *Z*-stacks were combined to a maximum projection. A line was drawn across the nucleus, and its plot profile was measured by ImageJ and normalized in relation to total values for AB414 and DAPI along the line.

To quantify the occupancy of DNA distribution in nurse cell nuclei in two dimensions (FISH experiments), we used a macro written for Image Pro Plus (D. Kelly, Wellcome Trust Centre for Cell Biology) to measure the total nuclear area on the DAPI images (manually intensity-thresholded and background-subtracted) and then divided the DAPI-defined nucleus into five shells of equal area. The percentage of DAPI signal was estimated in the outermost shell, which occupied 20% in area or ∼10% of the nuclear radius. For the FISH probes, we took measurements for the three most central *Z*-stacks of every nucleus. The average of three lines from the center to the Lamin signal was used as the radius of each nucleus. Positions of FISH signals were measured as the shortest distance to the nearest point of the Lamin staining, and the proportion of the signals within 10% of the nuclear radius from the periphery was estimated.

To measure the area occupied by chromatin in S2 cell nuclei, we used DAPI and Lamin immunostaining images taken using the same setting and subtracted the background (ImageJ). The DAPI images were manually thresholded to the highest background value in the cytoplasm, and areas were measured with a particle size setting of 9 to infinity. Total area values for DAPI were calculated relative to total nuclear area defined by Lamin.

The diameters of the oocyte nuclei were measured by choosing the mid-*Z*-plane of the nucleus and averaging two measurements of the nuclear diameter in a 90° angle to each other. The signal intensity of AB414 and Nup62 at the nuclear envelope was determined on a maximum projection image of three *Z*-stacks using the formula (*I*_NE_ − *I*_cyt_)/*I*_cyt_, where *I*_cyt_ and *I*_NE_ were defined as the averages of each maximum intensity in three boxes with a defined size in the cytoplasm (background) and on the nuclear envelope (signal), respectively.

Fisher's exact test, Wilcoxon test, and *t*-test were used for categorical, nonparametric, and parametric data, respectively.

## Supplementary Material

Supplemental Material
