# Supplementary material for: A negative loop within the nuclear pore complex controls global chromatin organization
Source: Genes Dev. 2015 Sep 1;29(17):1789–94. doi: 10.1101/gad.264341.115 (PMC4573852; doi:10.1101/gad.264341.115)
Supplement: Supplemental Material [file supp_29_17_1789__index.html]

Supplemental Material 

# A negative loop within the nuclear pore complex controls global chromatin organization

## Supplemental Material

**Files in this Data Supplement:**

- Supplemental Figures.pdf
