# Supplementary material for: A negative loop within the nuclear pore complex controls global chromatin organization
Source: Genes Dev. 2015 Sep 1;29(17):1789–94. doi: 10.1101/gad.264341.115 (PMC4573852; doi:10.1101/gad.264341.115)
Supplement: Supplemental Material [file supp_29.17.1789_Supplemental_Figures.pdf]

Breuer_FigS1

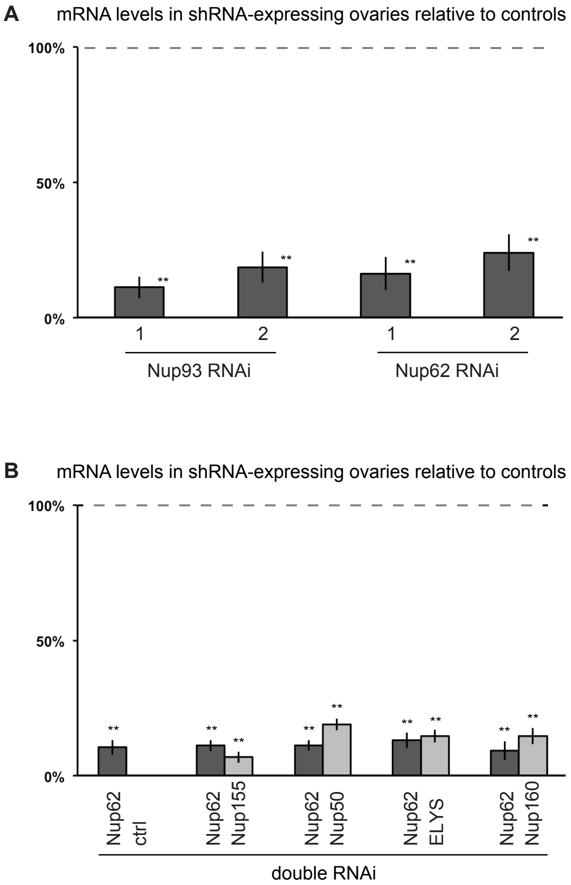

Figure S1, related to Fig1: mRNA is decreased in ovaries expressing shRNA
(A) The relative mRNA levels of target genes in ovaries expressing shRNA compared to control ovaries. Quantitative RT-PCR was used for the estimation. (B) The relative mRNA levels of Nup62 and another target gene in ovaries expressing two shRNA targeting Nup62 and the second gene compared to control ovaries. Error bars represent standard errors of the mean derived from technical triplicates.
** indicates a significant difference of the means from controls ($p<0.0001$).

Breuer_FigS2

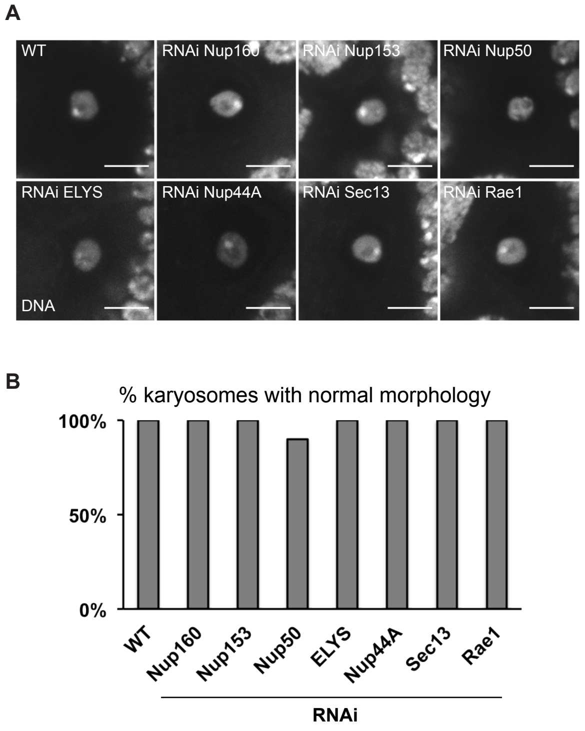

Figure S2, related to Fig1: Chromatin attachment phenotype by Nup62 or Nup93 RNAi is not a phenotype generally seen by RNAi of NPC subunits

(A) Representative DNA-stained images of karyosomes in oocytes expressing shRNA for NPC subunits. (B) Frequencies of karyosomes with spherical morphology. $5 \leq n \leq 12$. RNAi of other NPC subunits did not show a chromatin attachment phenotype similar to Nup62 or Nup93 RNAi. Bar=5 μm.

Breuer_FigS3

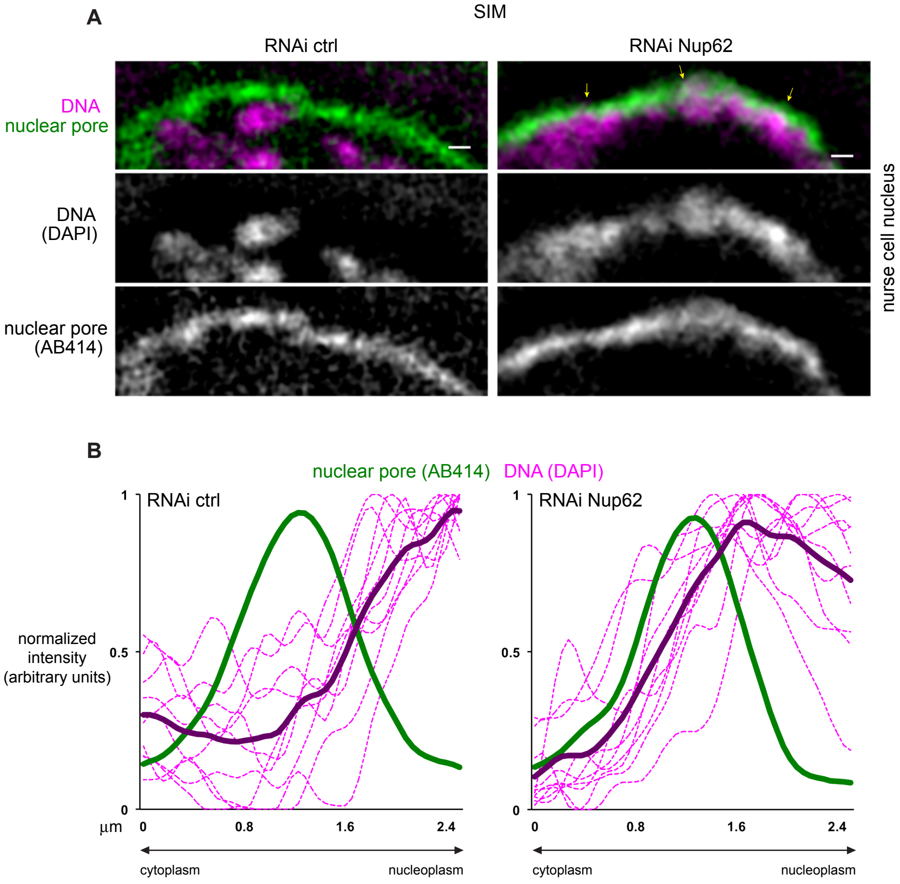

Figure S3, related to Fig1: Close proximity of chromatin to nuclear pores in Nup62 RNAi nurse cells
(A) Structural illumination microscopy (SIM) images of nurse cell nuclei stained with DAPI (DNA) and AB414 (nuclear pores). The arrows indicate overlaps between DNA and nuclear pore signals. Bar=1.0 µm. (B) Signal intensity along a line drawn perpendicular to the nuclear envelope of nurse cells. The mean intensity of nuclear pore signals along ten lines from three nuclei is shown by a thick green line. The mean and individual intensity of DNA signals along the same ten lines are shown by a thick dark purple line and thin dotted purple lines, respectively.

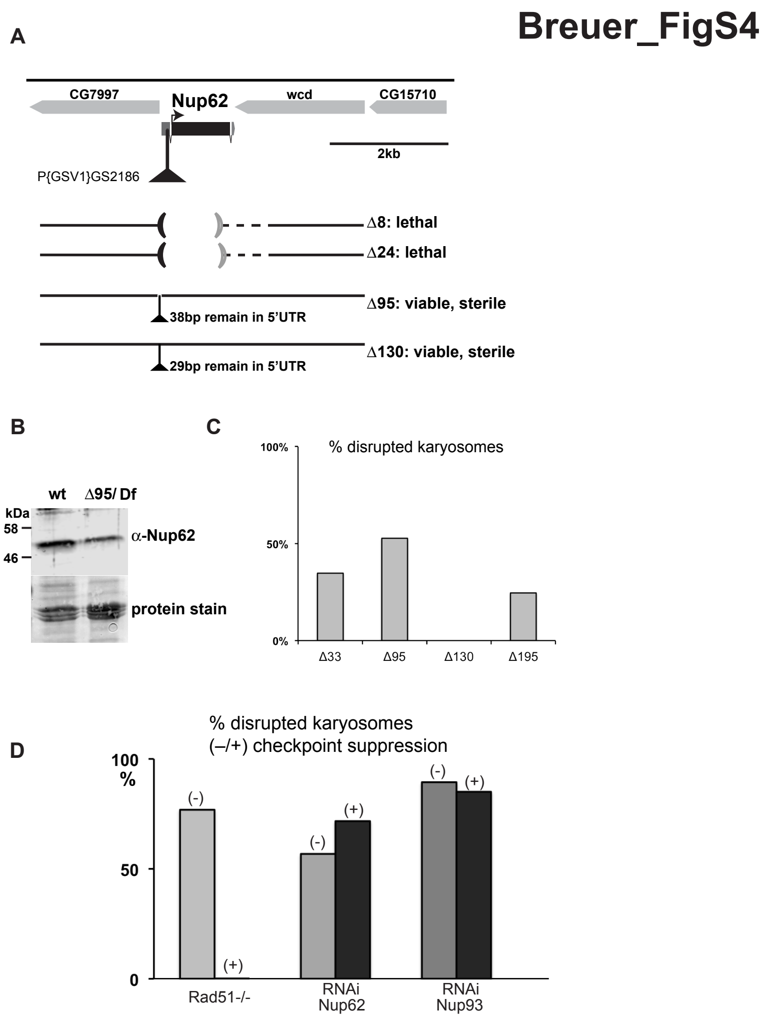

Figure S4, related to Fig1: Female sterile Nup62 mutants show karyosome defects, and the karyosome defects caused by Nup62 or Nup93 RNAi are independent of the meiotic recombination checkpoint
(A) The genomic region surrounding the Nup62 gene, indicating the position of P-element that was mobilised to generate Nup62 mutants, regions deleted in lethal alleles (Δ8 and Δ24) and residual P-element in viable, female sterile alleles (Δ95, Δ130). (B) Western Blot showing reduced protein levels compared to control in ovaries. (C) Frequencies of abnormal karyosomes in each of all four female sterile alleles. (D) The karyosome defects caused by loss of Nup93 or Nup62 is independent of the meiotic recombination checkpoint. Flies mutant for DmRad51 (spnA) or expressing shRNA for Nup93 or Nup62 were crossed to flies with a mutant allele of the checkpoint kinase DmChk2 (mnk) or adult females were fed with caffeine. Rad51-phenotype (–) is rescued upon suppression of the checkpoint (+), whereas the phenotype caused by Nup93 or Nup62 RNAi persists in the same conditions. As the effects of the chk2 mutation and caffeine feeding are similar, the results were pooled together.

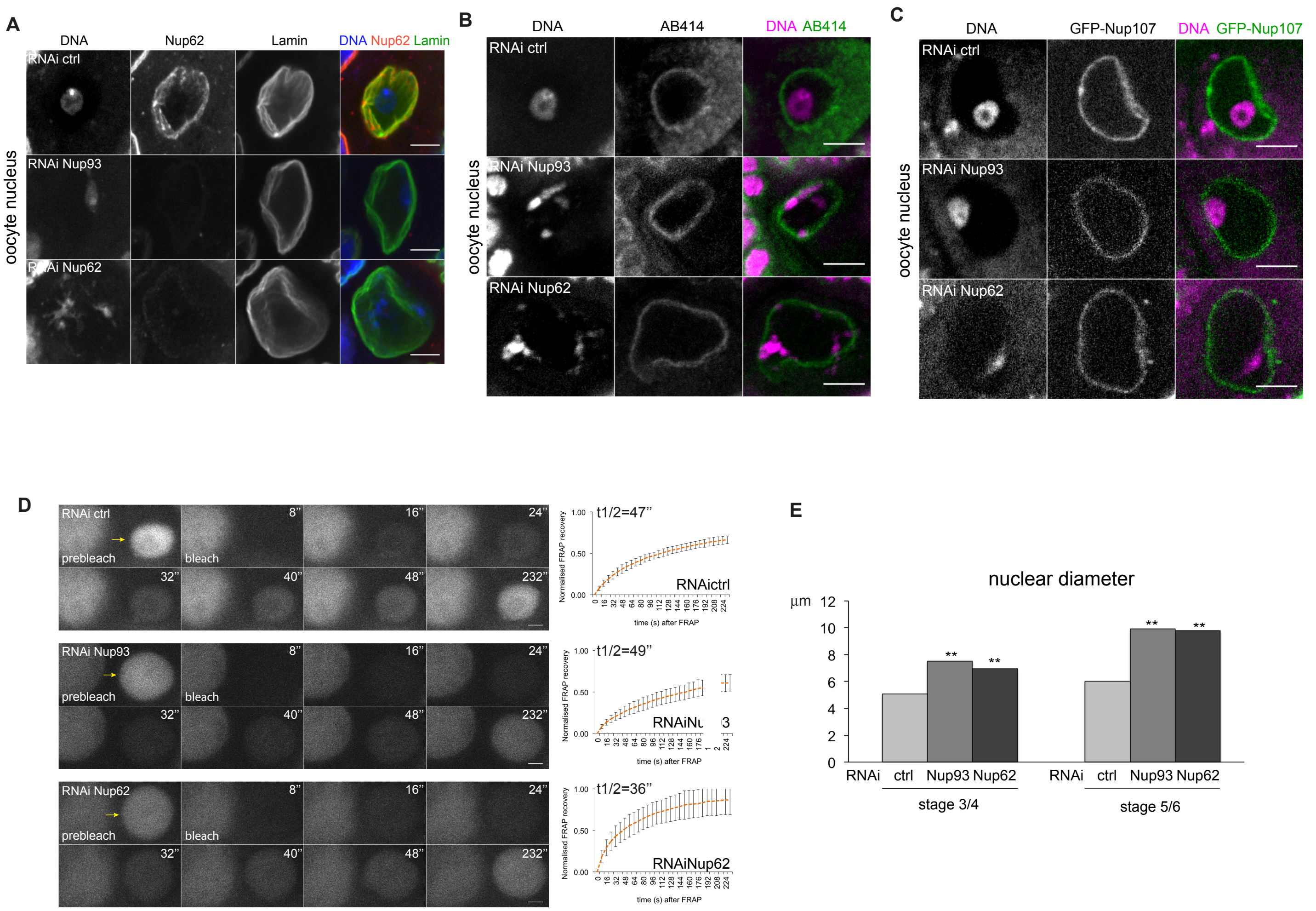

Figure S5, related to Fig2: Structural integrity and functional integrity for transport are largely intact
(A) Immunostaining shows a reduction of Nup62 protein from the nuclear envelope in ovaries expressing Nup93 or Nup62 shRNA compared to the ones expressing a control shRNA. Scale bar is 5 µm. (B) The localisation of FG-containing nuclear pore subunits detected by AB414 is unchanged in all RNAi conditions. Bar=5 µm. (C) GFP-Nup107 localisation is unchanged in ovaries expressing shRNA against a control, Nup93 or Nup62 together with GFP-Nup107. Bar=5 µm. (D) FRAP experiments of GFP-NLS in oocytes (right; arrows) expressing shRNA against a control, Nup93 or Nup62 as well as GFP-NLS. Area corresponding to nucleus was bleached and recovery monitored (see Material & Methods). Graphs depicting normalised recovery curve over time, half-life values for each experimental group indicated (control RNAi, n=17; Nup93 RNAi, n=17; Nup62 RNAi, n=23). Error bars indicate standard errors. (E) Nuclear size in oocytes is increased upon RNAi of Nup93 and Nup62. Diameter of oocyte nuclei was measured in control RNAi (n=39), Nup93 RNAi (n=27) and Nup62 RNAi (n=32) for developmental stages 3-4 and 5-6 (**p=0.0001).

Breuer_FigS6

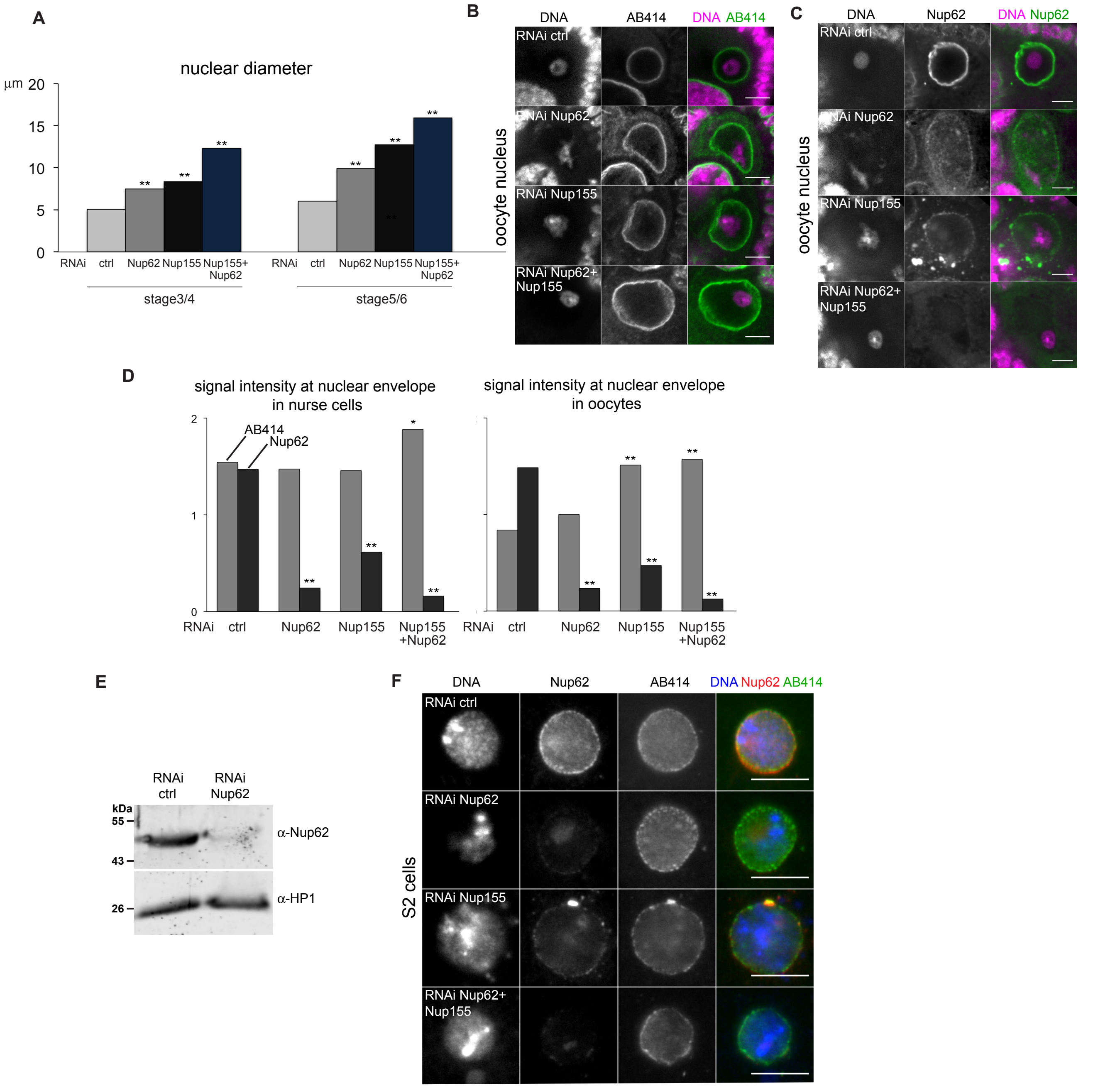

Figure S6, related to Figure 3 and 4: Nup62 is lost but other FG-containing subunits are retained after Nup62 or Nup155 RNAi
(A) The large nuclear size caused by Nup62 depletion is not rescued upon co-depletion of Nup155. The nuclear diameters were measured in oocytes expressing shRNA against control (n=39), Nup62 (n=33), Nup155 (n=29) or Nup62+Nup155 (n=28). (**p=0.0001). (B) Nuclear pore integrity is maintained after RNAi against Nup62 and Nup155. Oocytes expressing shRNA against control, Nup62, Nup155 or simultaneously Nup62+Nup155 were fixed and stained for FG-containing subunits using AB414 and DNA. AB414 staining was unchanged in all oocyte nuclei. Bar=5 µm. (C) Nup62 is reduced at the nuclear periphery upon Nup62 and Nup155 RNAi. Oocytes expressing shRNA against a control, Nup62, Nup155 or simultaneously Nup62+Nup155 were fixed and stained for Nup62 and DNA. Nup62 is strongly reduced from the nuclear envelope in all conditions except the control RNAi. In the case of Nup155 RNAi, foci of Nup62 accumulate in the cytoplasm. Bar=5 µm. (D) Intensity of AB414 and Nup62 signals in nurse cells and oocytes. The relative signal intensity at nuclear envelope over the cytoplasm ((Ine-Icyt)/Icyt) is plotted for control RNAi (n=17), Nup62 RNAi (n=14), Nup155 RNAi (n=16) and Nup62+Nup155 RNAi (n=20). (nurse cells *p=0.0412; oocytes **p=0.0003 and p=0.0006). (E) Nup62 is depleted after Nup62 RNAi. Western blot of protein extracts from S2 cells treated with control or Nup62 dsRNA, probed by anti-Nup62 and anti-HP1 antibodies. (F) Effect of Nup62 or Nup155 RNAi on the localisation of Nup62 and FG-containing subunits in S2 cells. Cells treated with dsRNA against control, Nup62, Nup155 or Nup62+Nup155. Cells were fixed and stained for Nup62, AB414 and DNA. Bar=5 µm.
